# Extensively Drug-resistant Salmonella typhi Meningitis in a 16-year-old Male

**DOI:** 10.7759/cureus.5961

**Published:** 2019-10-22

**Authors:** Noman Khurshid, Bilal Ahmed Khan, Syed Wasif Bukhari, Ashar Shahid, Avinash Punshi

**Affiliations:** 1 Miscellaneous, Dow Medical College, Dow University of Health Sciences, Karachi, PAK; 2 Internal Medicine, Dow Medical College, Dow University of Health Sciences, Karachi, PAK; 3 Internal Medicine, Dow University of Health Sciences, Karachi, PAK; 4 Internal Medicine, Civil Hospital Karachi, Karachi, PAK

**Keywords:** antibiotic resistance, culture, cephalosporin, meningitis, salmonella, typhi

## Abstract

Meningitis caused by Salmonella enteritidis is an uncommon infection, linked with great mortality and neurological problems, which makes prompt diagnosis and treatment very crucial. Patients of bacterial meningitis present with common symptoms such as headache, fever, altered level of consciousness, and neck rigidity. A positive gram stain or culture of cerebrospinal fluid (CSF) leads to its diagnosis. In this report, we present a case of a 16-year-old male with extensively drug-resistant (XDR) Salmonella typhi meningitis, which was not responsive to initial medical intervention. He was treated with meropenem, imipenem, azithromycin, and metronidazole. Immediate tracheostomy and intubation were performed in the surgical intensive care unit (ICU), as the patient had developed stridor, shortness of breath, tachypnea, tachycardia, and had severely decreased O_2_ saturation of 60%. As far as treatment is concerned, third-generation cephalosporins are considered the treatment of choice. In addition, the use of fluoroquinolones and carbapenems, mainly meropenem, has also been described as a therapeutic alternative.

## Introduction

The causative agent of Salmonella typhi meningitis is Salmonella, a gram-negative bacilli bacteria belonging to the family of Enterobacteriaceae [1]. Salmonella strains, being an important pathogen of childhood and infancy diseases, mostly cause acute gastroenteritis or typhoid, and the disease rarely presents as meningitis and bacteremia and often as brain abscess [2-3]. While being an uncommon disease with 1% cases in the developed world, Salmonella typhi meningitis affects around 13% of infants and children in the developing countries, with a mortality rate of around 90%, irrespective of the serotype [4].

Patients present with common symptoms such as headache, fever, altered level of consciousness, and neck rigidity. A positive gram stain or culture of the cerebrospinal fluid (CSF) leads to its diagnosis [5]. Improper hygiene, unsanitary food, and unsatisfactory vaccination coverage in most areas of Pakistan have played a major role in the spread of the disease.

In this report, we present a case of a 16-year-old male with extensively drug-resistant (XDR) Salmonella typhi meningitis, which was not responsive to initial medical intervention.

## Case presentation

A 16-year-old male patient came to the emergency department of Civil Hospital Karachi with a complaint of high-grade fever for 14 days, headache and neck pain for 12 days, vomiting for 10 days, and altered level of consciousness since one day. According to the patients' attendant, he was in his usual state of health 14 days ago, when he developed a high-grade fever that was sudden in onset, associated with rigors and chills, and didn't follow a certain pattern. However, it was partially relieved by taking medicine from a nearby clinic. The patient also complained of neck stiffness and pain for the past 12 days along with the fever and eight to nine episodes of vomiting per day for 10 days, which usually contained food particles and was non-projectile, without any odor or blood and approximately a glass full. Since the last one day, the patient also developed an altered level of consciousness gradually. There was also a history of an episode of a fit a day before. He gave a history of headache, neck pain, hemoptysis, and shortness of breath the same day. One of his close family members had tuberculosis (TB).

On examination, he had blood pressure (BP) of 130/80 mm of Hg, pulse rate of 102 beats/minute, a temperature of 100ºF, and a respiratory rate of 16 breaths/minute. His neck stiffness came out to be positive while Kernig's sign was also positive. The patient was irritable, didn't respond well, and was not well-oriented. His plantars were bilaterally downgoing.

The plan was to do magnetic resonance imaging (MRI), 24 hourly urinary protein, lumbar puncture, and random blood sugar every four hours. His WBC count was 5500 mm^3^, hemoglobin concentration was 9.3 g/dl, and platelet count was 185,000 mm^3^. His C-reactive protein level was 14.6 mg/dl. Urine D/R showed protein 2++, glucose 2++, ketone bodies 2++, blood 2++, and RBCs about 20-25 cells. CSF fluid examination revealed numerous pus cells. The result for gram-negative rods came negative with Indian ink. The culture had moderate growth of Salmonella typhi that was resistant to all drugs but was susceptible to meropenem, azithromycin, and imipenem and thus showed extended drug resistance.

**Figure 1:**
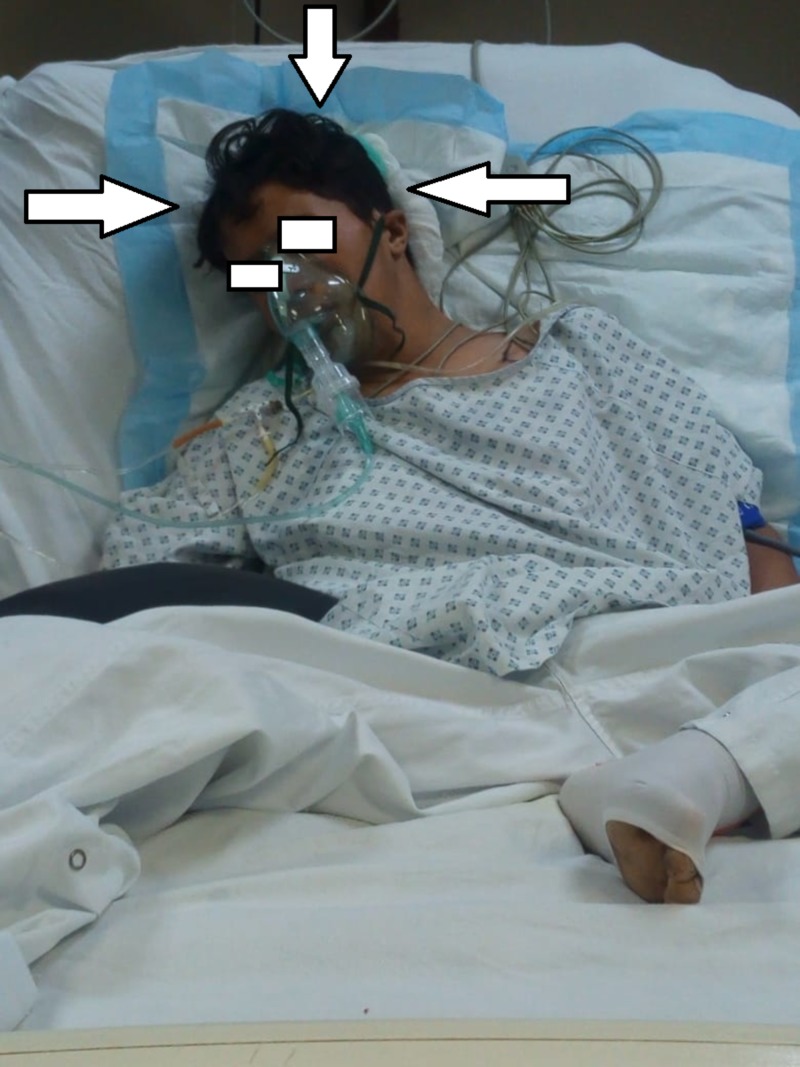
Patient admitted in the surgical ICU, Civil Hospital, Karachi ICU: intensive care unit

He was shifted to the surgical ICU (Figure 1), where they reported that a neck swelling had developed and the patient presented with stridor, shortness of breath, tachypnea, tachycardia, and O_2 _saturation of 60%.

**Figure 2:**
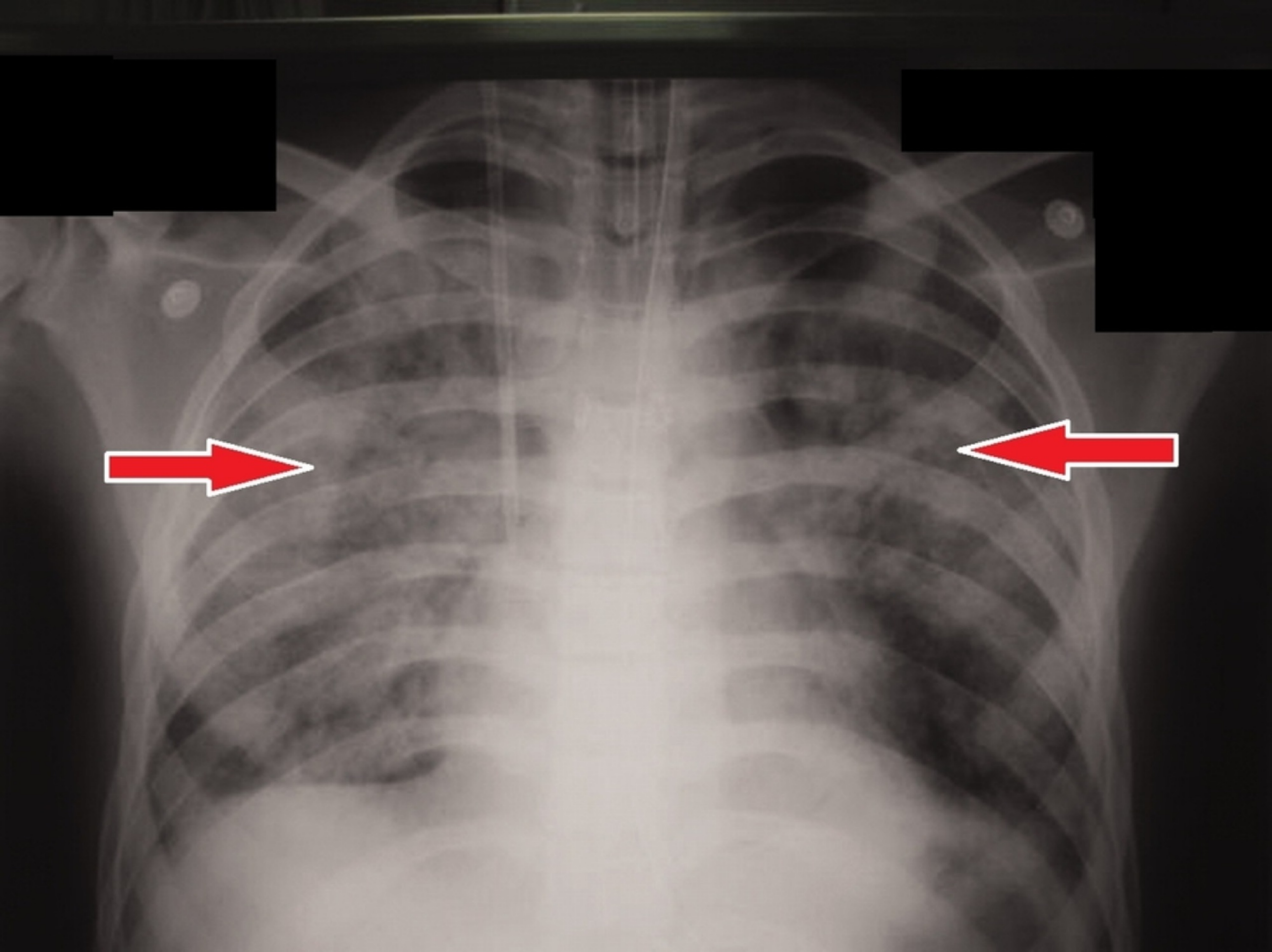
Chest X-ray

Immediate tracheostomy and intubation were performed and the ear, nose, throat (ENT) surgeon was also called. The plan was to give meropenem and clindamycin as gram-negatives. As metronidazole has better anaerobic coverage and is less resistant then clindamycin, metronidazole was given instead. The ENT specialist assessed the patient and noted him to be dyspneic, using accessory muscles for breathing, coarse crepitus bilaterally, harsh vesicular breathing, and an upper airway obstructed lesion. The chest X-ray showed pleural effusion bilaterally (Figure 2).

A chest tube was placed due to subcutaneous emphysema. The computed tomography (CT) scan showed dilated ventricles and mild cerebral edema. Symptoms improved with antibiotic use. The patients' health slowly progressed, his appetite and sleep were normal. Blood and stool cultures were found to be negative. He was admitted for the following 10 days and then later discharged when he was afebrile and feeling well. Meropenem was continued for a week, and the patient was advised to consult neurological therapy to reach neurodevelopmental goals.

## Discussion

Salmonella typhi meningitis is an uncommon presentation of salmonella infection. Since 1900, only around nine cases of Salmonella typhi meningitis in adults have been acknowledged, where most of these were stated in the pre-antibiotic times [6]. The New York Salmonella Centre studied 7779 infections; meningitis was identified in only 0.8% [7].

Most cases of Salmonella typhi meningitis are found in children less than one-year-old, predominantly in those less than three months old. If found in adults, it is associated with people who are immunocompromised or with underlying disorders such as human immunodeficiency virus (HIV) infection, malaria, or malnutrition. A high incidence of bacterial meningitis is documented from tropical areas but also infrequently from industrialized countries. The rate of mortality and the prevalence of neurological problems because of Salmonella meningitis are high, particularly in Africa.

Virulence factors like an intracellular invasion, polysaccharide capsule, and fimbriae possessed by S. typhi help it in colonizing the mucosa, surviving intravascularly, invading the meninges, and enduring in the subarachnoid space. The inflammatory processes and the immune complexes are stimulated in the host to bring these outcomes. S. typhi is most feared because of its intracranial infections, which may cause meningitis, subdural effusion, empyema, or brain abscess [8].

An observational study estimated that 13% of the patients died, 75% led to complications in the acute phase, such as hydrocephalus, subdural invasion, stroke, ventriculitis, cerebral empyema, and brain abscess, while some also showed cognitive delay and deterioration [9].

In a developing country like Pakistan, there is an uprising incidence of extensively drug-resistant (XDR) Salmonella typhi. However, after a thorough literature search, we found very few such cases reported globally. With the emergence of resistance to first-line drugs such as ampicillin, co-trimoxazole, and chloramphenicol, ciprofloxacin became the drug of choice for treating typhoid. Fluoroquinolones, as the empiric treatment of typhoid, have also been decreased due to the escalating resistance to ciprofloxacin testified by several regions. Drugs such as azithromycin or a third-generation cephalosporin were taken into account but, now, resistance to cephalosporin has been noted due to the production of extended-spectrum beta-lactamases (ESBLs) by the organism [10]. Carbapenems, principally meropenem, have also been labeled as a therapeutic substitute [11].

H58, a multidrug-resistant (MDR) haplotype of S. typhi has been spreading worldwide over the past 20 years, especially across South and Southeast Asia and parts of Africa and Oceania. Plasmid or transposon interchange facilitates antimicrobial resistance (AMR) genes transfer. Decreased vulnerability to fluoroquinolones is linked with chromosomal mutations and the attainment of AMR genes. In Pakistan, there is an emergence of MDR and quinolone-resistant S. typhi strains. Since then, the empirical treatment of choice in Pakistan for typhoid fever has been a third-generation cephalosporin such as ceftriaxone/cefotaxime (parenteral) or cefixime (oral). Since November 2016, a huge percentage of ceftriaxone-resistant cases have been reported from the cities of Hyderabad and Karachi in the province of Sindh, Pakistan. An analogous case was also recognized in the United Kingdom from a traveler returning from Pakistan [12]. In Pakistan, where there are no recognized guidelines on treating infectious diseases and no ban and little awareness on excessive over-the-counter antibiotics use, this is frightening, since it has made Pakistan the leading nation with emerging XDR cases.

As tuberculosis is so prevalent in our country, a diagnosis of tuberculosis meningitis was also entertained. But, to our surprise, blood culture revealed S. typhi and repeat CSF examination on the tenth day was normal without any anti-tuberculosis treatment.

Lastly, meningitis caused by Salmonella enteritidis is an infection that is unlikely to be seen but is linked with severe mortality and neurological complications, thus, making its prompt diagnosis and treatment very crucial.

## Conclusions

Salmonella typhi meningitis is a rare but distressing disease. It is, therefore, vital to consider bacterial meningitis high on the differential diagnosis for patients who present with altered mental status, even with atypical presentations. Early detection is essential to aid with disease management and decrease mortality.
